# hsa_circ_0004018 suppresses the progression of liver fibrosis through regulating the hsa-miR-660-3p/TEP1 axis

**DOI:** 10.18632/aging.103257

**Published:** 2020-06-25

**Authors:** Shan Li, Fangmin Song, Xu Lei, Jingtao Li, Fang Li, Huabing Tan

**Affiliations:** 1 Department of Infectious Diseases and Lab of Liver Disease, Renmin Hospital, Hubei University of Medicine, Shiyan, Hubei, China; 2 Department of Infectious Diseases, People’s Hospital of Yunxi, Shiyan, Hubei, China; 3 Department of Liver Diseases, The Affiliated Hospital of Shaanxi University of Chinese Medicine, Xianyang, Shaanxi, China

**Keywords:** liver fibrosis, hepatic stellate cells, circular RNA, microRNA

## Abstract

Efforts have been made in the prevention and treatment of liver fibrosis. The inhibition or depletion of the hepatic stellate cells (HSCs) has been considered as a potential approach. Recently, there are numbers of studies about the role of the circular RNA in the disease progression. However, the role of circular RNA in the regulation of HSCs and the progression of liver fibrosis remained elusive. In this study, we constructed a CCl4-induced liver fibrosis mouse model and overexpressed hsa_circ_0004018 in HSCs. Then, salvianolic acid B was used to treat HSCs *in vitro*. We found that hsa_circ_0004018 is downregulated in liver fibrogenesis. Luciferase reporter assay was performed to verify the interaction of hsa_circ_0004018, hsa-miR-660-3p and TEP1. It showed that hsa_circ_0004018 may act as a sponge of hsa-miR-660-3p, which can target and downregulate the expression of TEP1. hsa_circ_0004018 expressing lentivirus was used to investigate the *in-vivo* function of hsa_circ_0004018 in CCl4-induced liver fibrosis mice. We also reveal that the hsa_circ_0004018/hsa-miR-660-3p/TEP1 axis contributes to the proliferation and activation of HSCs. In addition, the overexpression of hsa_circ_0004018 alleviated the progression of liver fibrosis. In conclusion, our study highlights hsa_circ_0004018 as a potential biomarker and therapeutic target for liver fibrosis.

## INTRODUCTION

Liver fibrosis is a common feature of chronic liver injury caused by viral hepatitis, metabolic disorder, autoimmune conditions and other reasons [1]. It represents an early stage of liver cirrhosis which may develop into liver failure or liver cancer especially hepatocellular carcinoma [2]. The liver cirrhosis is estimated to lead to over 1 million deaths globally every year [3], raising it a challenge in preventing and treating the liver fibrosis at the early stage.

It has been well known that the hepatic stellate cells (HSCs) are the main source of fibrogenic cells and play a key role in the liver fibrogenesis [3, 4]. The HSCs reside in the perisinusoidal space between hepatocytes and sinusoidal endothelial cells and act as a reserve for vitamin A in normal condition [2]. Upon liver injury, the quiescent HSCs would be activated by the cytokines, such as interleukin-6 (IL-6) [5, 6], interleukin-17 (IL-17) [7–9] and interleukein-22 (IL-22) [8, 9], secreted from the neighboring cell types within the injured liver microenvironment, including Kupffer cells, hepatocytes, sinusoidal endothelial cells, leukocytes, et cetera [6]. The activated HSCs express amounts of α-smooth muscle actin (α-SMA) and secret collagens and extracellular matrix (ECM) abundantly to the liver tissue interspace, resulting in liver architecture remodeling [2, 4]. As the HSCs account for 80% of total type I collagen (COL1A1) in the fibrotic liver, the HSCs inhibition with drugs like salvianolic acid B (Sal B) is always considered as a potential therapy for liver fibrosis [2, 10, 11].

In the most recent years, a large number of studies have been done about the role of non-coding RNA (ncRNA), ranging from microRNA, long non-coding RNA (lncRNA) to circular RNA (circRNA), in the liver fibrosis and HSC activation [12–14]. Generally, the microRNAs function through interacting with the complementary mRNA targets, resulting in the degradation of the target mRNAs and the downregulation of the target genes that contribute to liver fibrosis, such as TGF-β, PTEN, β-Catenin, et cetera [14]. The functions of lncRNA in the liver fibrosis and the activation of HSC seem more complex [13]. Some lncRNAs (such as MALAT1, HOTAIR, et cetera) can sponge with some microRNAs and therefore indirectly regulate the expression of their downstream target genes to regulate the process of liver fibrosis [13]. In addition, some lncRNAs (such as MALAT1 and lnc-LFAR1) can regulate the function of HSCs via interacting with the key proteins of the TGF-β signaling pathway [13]. In contrast, the roles of circRNAs in liver fibrosis and the function of HSCs remain largely unknown, although it it has been identified for a long history [15]. The classical functional mechanism of circRNAs is to interact with microRNA or other molecules to regulate the gene expression in the transcript level [16].

In our previous study, we used microarray to explore the circRNA expression profile in the fibrotic livers isolated from the mouse model (data not published), and found that hsa_circ_0004018 has a significant low expression in the fibrotic livers. There are few studies about the function of hsa_circ_0004018. A previous clinical study indicated that hsa_circ_0004018 might associate with the progression of hepatocellular carcinoma (HCC), but the mechanisms remained unclear [17–19]. As known, the liver fibrosis is closely associated with the progression of HCC. Without proper treatment, liver fibrosis can eventually develop into cirrhosis and HCC [20]. We hypothesize that hsa_circ_0004018 may contribute to the progression of HCC through directly influencing the progression of liver fibrosis. This motivates us to further reveal the role of hsa_circ_0004018 in the progression of liver fibrosis.

## RESULTS

### The low expression of hsa_circ_0004018 especially in HSCs was associated with the poor progression of liver fibrosis in mouse model

**Figure 1:**
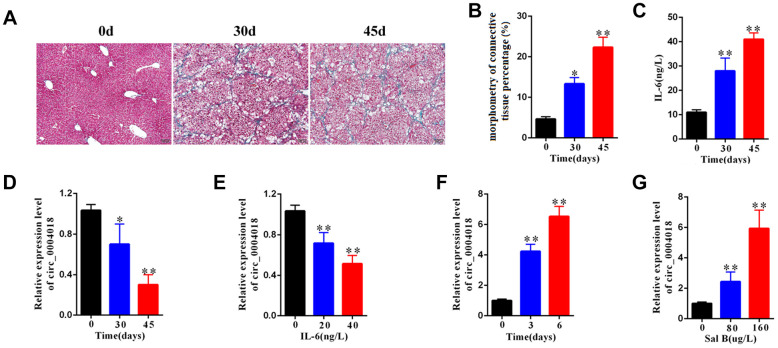
**The expression level of hsa_circ_0004018 was downregulated in mouse liver fibrosis model.** (**A**) The representative Masson’s trichrome staining of the liver samples isolated from the liver fibrosis mice sacrificed on day 0, 30 and 45 upon injection with CCl_4_. Five mice were sacrificed at each time point. (**B**) The percentages of fibrosis area of the liver samples isolated from the liver fibrosis mice sacrificed on day 0, 30 and 45 were counted through analyzing the proportion of the blue staining area by Image J software. Three visual field of each sample were analyzed. (**C**) IL-6 level in serum of the liver fibrosis mice sacrificed on day 0, 30 and 45 upon injection with CCl_4_. (**D**) The relative expression levels of hsa_circ_0004018 of the indicated liver samples were detected by real-time PCR. (**E**) The relative expressions levels of hsa_circ_0004018 of the primary HSCs treated with IL-6 at 0, 20 and 40 ng/L for 24 hours. (**F**) The relative expressions levels of hsa_circ_0004018 of the primary HSCs treated with Sal B (100ug/L) for 0, 2 and 5 days. (**G**) The relative expression levels of hsa_circ_0004018 of the primary HSCs treated with 0, 80 and 160ug/L Sal B respectively.

To examine the expression of hsa_circ_0004018 in the progression of liver fibrosis, we obtained the liver samples from the mice injected with CCl_4_ for 0, 30 and 45 days and detected the relative RNA levels of hsa_circ_0004018 by real-time PCR. We found that the severity of liver fibrosis and the serum level of IL-6 both increased over time upon CCl_4_ injection (Figure 1A–1C), while the relative expression of hsa_circ_0004018 was downregulated (Figure 1D). And we further examined the expression of hsa_circ_0004018 in IL-6 treated HSCs, and found that the relative RNA levels of hsa_circ_0004018 were significantly downregulated as the HSCs were treated with IL-6 at 0, 20 and 40 ng/L for 24 hours (p<0.01, Figure 1E)

It has been reported that salvianolic acid B (Sal B) can inhibit the progression of liver fibrosis through regulating the activation of HSCs [21–23]. We further examined the expression of hsa_circ_0004018 in Sal B treated HSCs, and found that the relative RNA levels of hsa_circ_0004018 were significantly upregulated over time as the HSCs were treated with 100 ug/L of Sal B for 0, 3 and 6 days (p<0.01, Figure 1F). In addition, the relative RNA levels of hsa_circ_0004018 significantly enhanced with the increase of Sal B concentration (p<0.01, Figure 1G), suggesting that the low expression of hsa_circ_0004018, especially in HSCs, was associated with the poor progression of liver fibrosis.

### Overexpression of hsa_circ_0004018 suppressed the proliferation and activation of HSCs *in vitro*

**Figure 2:**
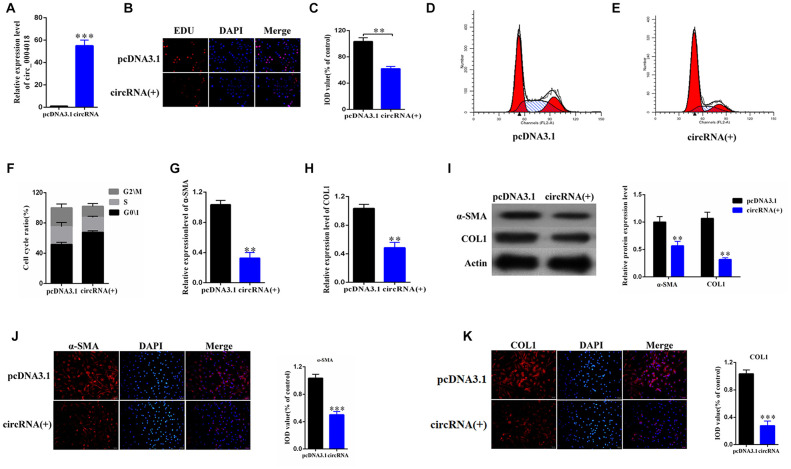
**The overexpression of hsa_circ_0004018 promoted G0/1 cell cycle arrest of the primary HSCs through upregulating the expression of α-SMA and COL1A1.** (**A**) The relative expression levels of hsa_circ_0004018 were detected by real-time PCR in the overexpressed HSCs. The vector was performed as control. (**B**, **C**) The EDU incorporating levels were measured in the hsa_circ_0004018 overexpressed HSCs and the control cells (**B**). The relative IOD values of the EDU incorporating of the indicated cells were statistically analyzed and presented as columns (**C**). Each group was independently examined three times. (**D**–**F**) The representative cell cycle distributions of the hsa_circ_0004018 overexpressed HSCs (**E**) and the control cells (**D**) were analysis by FACS. The cell distribution ratios of the G0/1 phase, the S phase and the G2/M phase of the indicated cells were statistically analyzed (**F**). (**G**, **H**) The relative RNA levels of α-SMA (**G**) and COL1A1 (**H**) of the hsa_circ_0004018 overexpressed HSCs and the control cells were detected by real-time PCR. (**I**) The expression of α-SMA and COL1A1 was detected by western blotting. (**J**) The expression of α-SMA was detected by immunofluorescence. (**K**) The expression of COL1A1 was detected by immunofluorescence.

To further study the role of hsa_circ_0004018 in HSCs, we overexpressed hsa_circ_0004018 in the primary HSCs (Figure 2A) and examined the proliferation of HSCs through the EDU incorporation experiment (Figure 2B). We found that the EDU incorporation levels of hsa_circ_0004018 overexpressed HSCs were lower than the vector control (Figure 2B, 2C). We further analyzed the effect of hsa_circ_0004018 overexpression on cell cycle, and found that the upregulation of hsa_circ_0004018 resulted in higher G0/1 phase ratio compared with the vector control (p<0.01, Figure 2D–2F), indicating that overexpression of hsa_circ_0004018 could suppress the proliferation of HSCs through promoting the cell cycle arrest.

In addition, we examined the expression of α-SMA and COL1A1, which reflect the activation of HSCs, in the hsa_circ_0004018 overexpressed and the control HSCs. We found that both RNA levels and protein levels of α-SMA and COL1A1 were downregulated upon overexpression of hsa_circ_0004018 comparing with the control cells (Figure 2G–2K), suggesting that the overexpression of hsa_circ_0004018 inhibited the activation of the primary HSCs *in vitro*.

### The hsa_circ_0004018 acts as a sponge of hsa-miR-660-3p

**Figure 3:**
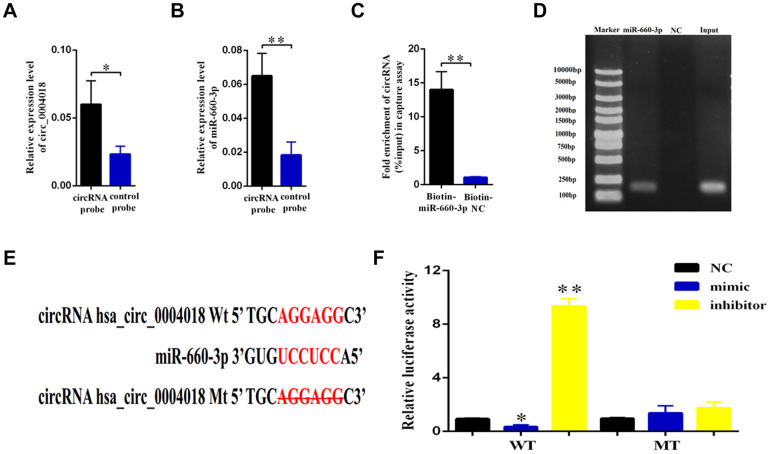
**Validation of hsa-miR-660-3p as the target of hsa_circ_0004018.** (**A**, **B**) HSCs lysis was pulled down with hsa_circ_0004018 specific probe, and then hsa_circ_0004018 (**A**) and hsa-miR-660-3p (**B**) were respectively detected by real-time PCR. (**C**, **D**) The HSCs were transfected with biotin labeled hsa-miR-660-3p and the negative control (NC) following by strepavidin enrichment, and then hsa_circ_0004018 was detected by real-time PCR. (**E**) The sequences between hsa_circ_0004018 and hsa-miR-660-3p were compared, and the complementary bases of red color indicate the seed sequence of hsa-miR-660-3p. The mutant hsa_circ_0004018 was designed without the seed sequence. (**F**) The HSCs stably expressing the luciferase construct containing the wild-type (WT) or mutant (MT) hsa_circ_0004018 were respectively transfected with hsa-miR-660-3p mimic, inhibitor and the negative control, and then the luciferase activity was examined.

The role of circular RNA as a sponge in regulation of microRNA has been well known [16]. In order to find out the potential target of hsa_circ_0004018, we used the Arraystar’s home-made microRNA target prediction software to scan globally the matching microRNA. We found that hsa_circ_0004018 shares microRNA response elements (MREs) of hsa-miR-660-3p (Figure 3E), which may interact with hsa_circ_0004018 in HSCs.

To confirm this hypothesis, we designed the biotin labeled hsa_circ_0004018 probe to detect the interaction in the primary HSCs. As expected, the hsa_circ_0004018 probe enriched more hsa_circ_0004018 (p<0.05, Figure 3A), as well as the hsa-miR-660-3p (p<0.01, Figure 3B), than the negative control probe. Consistently, the biotin labeled hsa-miR-660-3p enriched more hsa_circ_0004018 than the negative control probe (Figure 3C, 3D). To further explore the function of this interaction, the luciferase plasmids containing the wild type 3’ terminal (WT) or the MRE deleting 3’ terminal (MT) of hsa_circ_0004018 were constructed (Figure 3E) and simultaneously co-transfected with hsa-miR-660-3p mimic, negative control or inhibitor into the HSCs. After that, the luciferase activity of each combination was detected. It showed that the hsa-miR-660-3p mimic significantly suppressed the luciferase activity of WT (p<0.05, Figure 3F, Column 2) but not MT (Figure 3F, Column 5). Conversely, the hsa-miR-660-3p inhibitor significantly increased the luciferase activity of WT (p<0.01, Figure 3F, Column 3) but not MT (Figure 3F, Column 6), indicating that hsa_circ_0004018 functions as a sponge of hsa-miR-660-3p in HSCs.

### The hsa-miR-660-3p can promote the proliferation and activation of HSCs *in vitro*, and counteract with the function of hsa_circ_0004018

**Figure 4:**
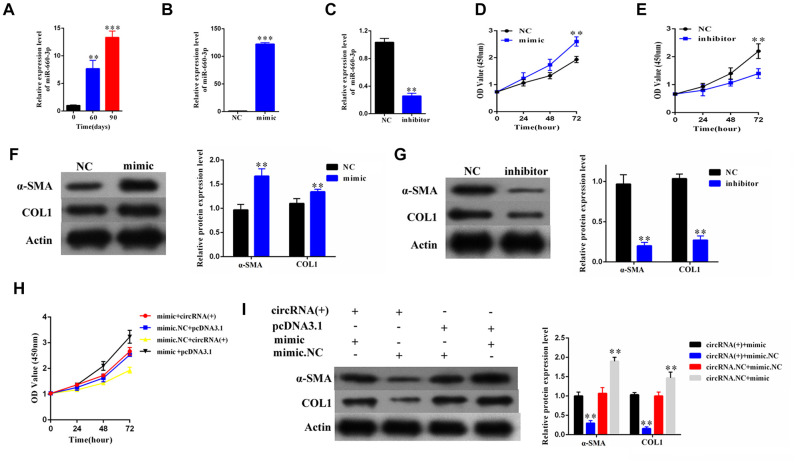
**The hsa-miR-660-3p can promote the proliferation and activation of HSCs *in vitro*, and counteract with the function of hsa_circ_0004018.** (**A**) It was detected the relative expression of hsa-miR-660-3p in the liver samples from CCl_4_-induced liver fibrosis mice sacrificed at 0, 60 and 90 days after CCl_4_ injection. (**B**, **C**) After transfection with mimic hsa-miR-660-3p (**B**) and inhibitor (**C**), the relative expression levels of hsa-miR-660-3p in HSCs were detected by real-time PCR. (**D**, **E**) After transfection with mimic hsa-miR-660-3p (**D**) and inhibitor (**E**), the growth curves of the HSCs were examined by CCK8 assay. (**F**, **G**) After transfection with mimic hsa-miR-660-3p (**F**) and inhibitor (**G**), the expression of α-SMA and COL1A1 in HSCs was detected by western blotting. (**H**, **I**) After transfection with the indicated mimic hsa-miR-660-3p or negative control (NC) plus hsa_circ_0004018 or the vector control, the growth curves of the HSCs were examined by CCK8 assay (**H**), while the expression of α-SMA and COL1A1 was detected by western blotting.

To explore the role of hsa-miR-660-3p in the HSCs and the progression of liver fibrosis, we firstly examined the expression of hsa-miR-660-3p in the primary HSCs isolated from the CCl_4_-induced liver fibrosis mice. It showed that the expression levels of hsa-miR-660-3p significantly increased over time upon CCl_4_ injection (p<0.01 at Day 60 and p<0.001 at Day 90, Figure 4A). The transfection of hsa-miR-660-3p mimic significantly promoted the proliferation of HSCs (p<0.01, Figure 4B, 4D), while the transfection of inhibitor showed the inhibitory effect (p<0.01, Figure 4C, 4E). In addition, the expression of α-SMA and COL1A1 significantly increased after transfection with mimic in HSCs (Figure 4F), whereas decreased after transfection with inhibitor (Figure 4G).

We then co-transfected the mimic with hsa_circ_ 0004018 in the HSCs and examined the proliferation and activation effect. As control, the negative control or the vector was transfected in combination as indicated (Figure 4H, 4I). The HSCs co-transfected with mimic and the vector control exhibited the fastest proliferation rate (black line, Figure 4H). In compare, the proliferation of HSCs co-transfected with mimic and hsa_circ_ 0004018 was significantly slower than the mimic/vector combination (red line vs. black line, Figure 4H), but faster than the negative control/ hsa_circ_0004018 combination (red line vs. yellow line, Figure 4H). We also examine the activation of the co-transfection HSCs, and found that the expression levels of α-SMA and COL1A1 were significantly lower in the mimic/ hsa_circ_0004018 combination than the mimic/vector combination (Lane 1 vs. Lane 4, Figure 4I), but higher than the negative control/hsa_circ_0004018 combination (Lane 1 vs. Lane 2, Figure 4I), indicating that hsa_ circ_0004018 counteract with the function of hsa-miR-660-3p.

### TEP1 was validated as a bona fide target of hsa-miR-660-3p

**Figure 5:**
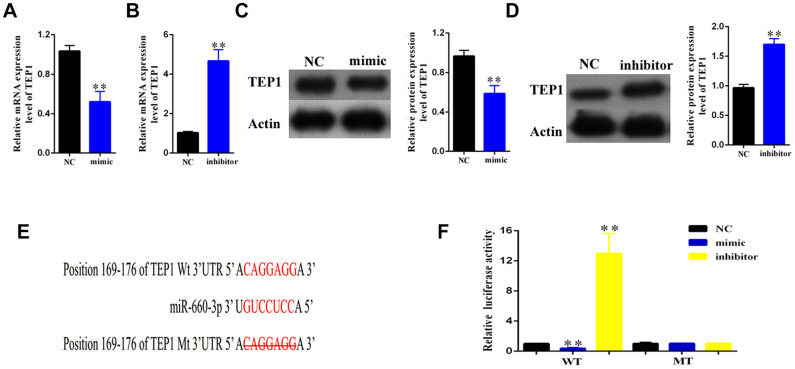
**Validation of TEP1 as a bona fide target of hsa-miR-660-3p.** (**A**, **B**) After transfection with mimic hsa-miR-660-3p (**A**) and inhibitor (**B**), the RNA levels of TEP1 were detected by real-time PCR. (**C**, **D**) After transfection with mimic hsa-miR-660-3p (**C**) and inhibitor (**D**), the expression of TEP1 was detected by western blotting. (**E**) The sequences between TEP1 3’UTR and hsa-miR-660-3p were compared, and the complementary bases of red color indicate the seed sequence of hsa-miR-660-3p. The mutant TEP1 3’UTR was designed without the seed sequence. (**F**) The HSCs stably expressing the luciferase construct containing the wild-type (WT) or mutant (MT) TEP1 3’UTR were respectively transfected with hsa-miR-660-3p mimic, inhibitor and the negative control, and then the luciferase activity were examined.

To find out the downstream target of hsa-miR- 660-3p, we used the miRBase for screening (http://www.mirbase.org/). As standard, the potential gene target should harbor a similar MRE with the seed sequence of hsa-miR-660-3p and hsa_circ_0004018. Accordingly, we finally focused on TEP1. To confirm this, we transfected the HSCs with hsa-miR-660-3p mimic or inhibitor, and examined the expression of TEP1. It showed that the transfection of mimic resulted in an obvious decrease in both RNA and protein levels of TEP1 (Figure 5A, 5C). In contrast, the transfection of inhibitor resulted in an obvious increase in both RNA and protein levels of TEP1 (Figure 5B, 5D).

In further, we constructed the luciferase plasmids containing the wild type 3’ UTR (WT-3’UTR) or the MRE deleting 3’ UTR mutant (MT-3’UTR) of TEP1 (Figure 5E). Either of these two plasmids was co-transfected with hsa-miR-660-3p mimic, negative control or inhibitor in the HSCs, and then the luciferase activity was examined. It showed that the hsa-miR-660-3p mimic significantly suppressed the luciferase activity of WT-3’UTR (p<0.01, Figure 5F, Column 2) but not MT-3’UTR (Figure 5F, Column 5). In contrast, the hsa-miR-660-3p inhibitor significantly increased the luciferase activity of WT-3’UTR (p<0.01, Figure 5F, Column 3) but not MT-3’UTR (Figure 5F, Column 6), indicating that TEP1 is a bona fide target of hsa-miR-660-3p in HSCs.

### Overexpression of hsa_circ_0004018 increased the expression of TEP1, which can significantly suppress the proliferation and activation of HSCs

**Figure 6:**
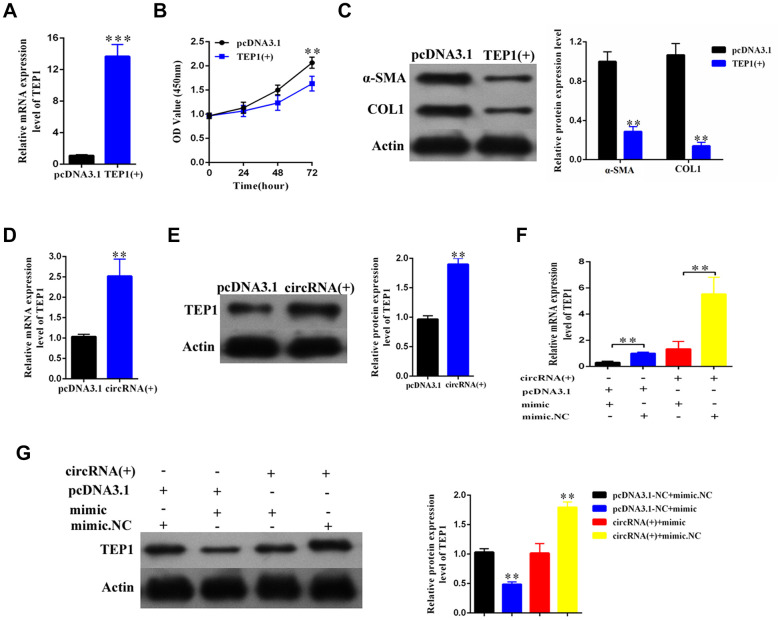
**The hsa_circ_0004018 suppressed the proliferation and activation of HSCs through upregulating the expression of TEP1.** (**A**) After transfection with TEP1 containing plasmid and the vector control, the RNA levels of TEP1 were detected by real-time PCR. The data are from three independent experiments. (**B**, **C**) After overexpression with TEP1, the growth curves of the HSCs were examined by CCK8 assay (**B**), while the expression of α-SMA and COL1A1 in HSCs was detected by western blotting (**C**). (**D**, **E**) The RNA levels (**D**) and the protein levels (**E**) were respectively detected after overexpression with hsa_circ_0004018. The vector was transfected as control. (**F**, **G**) After transfection with the indicated mimic hsa-miR-660-3p or negative control (NC) plus hsa_circ_0004018 or the vector control, the RNA levels (**F**) and the protein levels (**G**) of TEP1 in HSCs were respectively detected by real-time PCR and western blotting.

To explore the role of TEP1, we overexpressed TEP1 in the primary HSCs (Figure 6A). We found that the upregulation of TEP1 significantly inhibited the proliferation of HSCs (p<0.01, Figure 6B), and resulted in an obvious decrease in the expression of α-SMA and COL1A1 (Figure 6C).

In addition, both the RNA and protein levels of TEP1 significantly increased after overexpression of hsa_ circ_0004018 in the HSCs (Figure 6D, 6E). We further co-transfected the mimic with hsa_circ_0004018 in the HSCs and then examined the expression of TEP1. As control, the negative control or the vector was transfected in combination as indicated (Figure 6F, 6G). As expected, both the RNA and protein levels of TEP1 of the mimic/vector combination were significantly lower than the negative control/vector combination (p<0.01, Figure 6F, Column 1 vs. Column 2; Figure 6G, Lane 2 vs. Lane 1). In parallel, the RNA and protein levels of TEP1 of the mimic/hsa_circ_0004018 combination were significantly lower than the negative control/ hsa_circ_0004018 combination (p<0.01, Figure 6F, Column 3 vs. Column 4; Figure 6G, Lane 3 vs. Lane 4), indicating that hsa_circ_0004018 and hsa-miR-660-3p counteract with each other in the regulation of TEP1.

### Overexpression of hsa_circ_0004018 *in vivo* alleviated the progression of CCl_4_-induced liver fibrosis in mouse model

**Figure 7:**
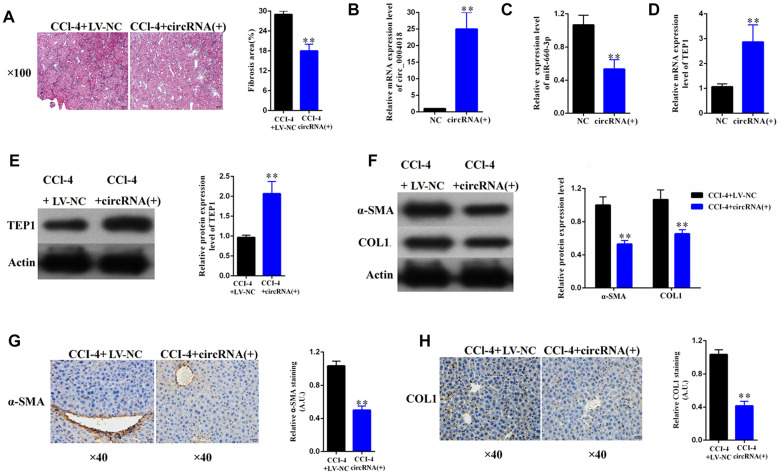
**Overexpression of hsa_circ_0004018 alleviated CCl_4_-induced mouse liver fibrosis through suppressing the expression of hsa-miR-660-3p and upregulating TEP1 in HSCs.** (**A**) The representative section staining of the liver samples isolated from the CCl_4_-induced liver fibrosis mice respectively injected with lentivirus expressing hsa_circ_0004018 (cirRNA group) or the negative control (NC group). (**B**, **C**) They were detected by real-time PCR the RNA levels of hsa_circ_0004018 (**B**) and hsa-miR-660-3p (**C**) in the liver samples of the cirRNA group and the NC group mice. (**D**, **E**) The RNA levels (**D**) and the protein levels of TEP1 (**E**) were respectively detected in the liver samples of the cirRNA group and the NC group mice. (**F**–**H**) It was detected the expression of α-SMA and COL1A1 in liver samples of the cirRNA group and the NC group by western blotting (**F**) and immunohistochemistry (**G**, **H**), respectively.

To explore the therapeutic potential of hsa_circ_ 0004018 overexpression, we further transduced the hsa_circ_0004018 expressing lentivirus into the livers of CCl_4_-induced liver fibrosis mice. It showed that liver fibrosis was alleviated in the hsa_circ_0004018 lentivirus transduced mice comparing with the control mice (Figure 7A, 7B). In accordance with the results of the *in-vitro* experiments, the relative levels of hsa-miR-660-3p were significantly lower in the hsa_circ_0004018 lentivirus transduced livers than the control livers (Figure 7C), while both the RNA and protein levels of TEP1 were significantly higher in the hsa_circ_0004018 lentivirus transduced livers than the control livers (Figure 7D, 7E). Meanwhile, the expression levels of α-SMA and COL1A1 obviously decreased after transduction with hsa_circ_0004018 lentivirus (Figure 7F–7H), suggesting that the upregulation of hsa_circ_ 0004018 suppressed the activation of HSCs *in vivo*.

## DISCUSSION

There are numbers of studies implying that circular RNA may participate in the progression of diseases including cancer, diabetes etc [15]. However, little is known about the role of circular RNA in liver fibrosis. In this study, we used the CCl_4_-induced mouse model to explore the role of hsa_circ_0004018 in liver fibrogenesis, and found that the low expression of hsa_circ_0004018 is associated with the poor progression of liver fibrosis (Figure 1). In the previous study, it has been found that the expression hsa_circ_0004018 in hepatocellular carcinoma (HCC) was significantly lower than para-tumorous tissue; in addition, the expression levels of hsa_circ_0004018 in the liver cirrhosis samples were significantly lower than HCC, exhibiting HCC-stage-specific characteristics [17]. As known, the liver fibrosis is an early stage feature of liver cirrhosis. It is possible that the continual downregulation of hsa_circ_0004018 significantly contributes to the transformation from liver fibrosis to HCC.

The function of circular RNA is not completely understood. Generally, circular RNA is considered to interact with microRNA and resist the function of microRNA through the microRNA response elements (MREs) [16]. Through bioinformatical screening, we further revealed that hsa_circ_0004018 acts as sponge of hsa-miR-660-3p, which can target and downregulate the expression of TEP1 (Figures 3, 5). Undoubtedly, there may exist other targets of hsa_circ_0004018 apart from hsa-miR-660-3p and other downstream targets of hsa-miR-660-3p apart from TEP1. Considering that hsa-miR-660-3p inhibitor and TEP1 overexpression reappear well the phenotypes of hsa_circ_0004018 downregulation (Figures 4, 6), we highlight the hsa_circ_0004018/hsa-miR-660-3p/TEP1 axis in the proliferation and activation of HSCs and liver fibrosis.

In our study, we found that telomerase-associated protein 1 (TEP1) is the downstream target of hsa-miR-660-3p. With the overexpression of hsa_circ_0004018, the expression of TEP1 was upregulated (Figure 6E). The telomeres are known to shorten with age and associated with increased incidence of chronic liver disease [24]. As a component of the telomerase, TEP1 can contribute to the maintenance of telomeres [25]. A number of studies have been reported that the hepatic or immune cell telomeres may associate with liver fibrosis [24]. However, the function of TEP1 in the HSCs and its role in liver fibrosis remained unknown before. In this study, we found that TEP1 overexpression suppressed the activation of HSCs *in vitro*, but the underlying mechanism still needs further study in the future.

To further test the therapeutic potential of hsa_circ_ 0004018, we transduced the hsa_circ_0004018 expressing lentivirus into the CCl_4_-induced liver fibrosis mice. It is exciting that hsa_circ_0004018 overexpression alleviated the progression of liver fibrosis through inhibiting the activation of HSCs (Figure 7). In conclusion, we identify here hsa_circ_0004018 as a promising biomarker and highlight hsa_circ_0004018 upregulation as a potential approach of the prevention and therapy for liver fibrosis.

## MATERIALS AND METHODS

### Plasmids

The sequence of hsa_circ_0004018 was obtained from CircBase (http://circbase.org, genome position: chr17: 1703150-1704318). The cDNA of hsa_circ_0004018 was cloned into the pcDNA3.1(+) circRNA mini vector with EcoRI and SacI restriction sites:

 (forward primer: 5’-GAATTCACACTCCAATTCTC TGCCTAC-3’;

 reverse primer: 5’-GAGCTCCAAGACCAGTCTGG GCAAC-3’),

and into the pLO-ciR vector with EcoRI and NdeI resctriction sites:

 (forward primer: 5’-GAATTCACACTCCAATTCTC TGCCTAC-3’;

 reverse primer: 5’-CATATGCAAGACCAGTCTGG GCAAC-3’).

The sequence of TEP1 was obtained from NCBI (NM_001319035). The cDNA was cloned into pcDNA3.1(+) vector with NotI and XbaI restriction sites:

 (forward primer: 5’-GCGGCCGCATGGAAAAACT CCATGGGC-3’;

 reverse primer: 5’-TCTAGATCATTCCCAATTCAG AAAGTAC-3’).

All the plasmids were sequenced before use.

### Cell culture

The isolation of HSCs was referred to the previous protocol [26, 27]. Firstly, we carefully isolated the livers from the normal mice or the liver fibrosis mice. Then we digested the livers with pronase E (0.4mg/ml, Sigma Aldrich, Catalog# 1074330001)/collagenase P (1.5mg/ml, Sigma Aldrich, Catalog#11213857001) solution to made disperse cells. After being sorted by FACS, the HSCs population was collected and seeded at a density of 2×10^4^ cells/cm^2^ in the culture plates with HSC culture medium (DMEM plus 10% FBS, 4mM L-glutamine and 1% penicillin-streptomycin solution) (DMEM: GIBCO, Catalog#11965; FBS: Biological Industry, Catalog#04-001-1A; L-glutamine: Sigma Aldrich, Catalog#G3126), and incubated in the incubator with 5% CO_2_ at 37 °C overnight. After attachment, the cells were carefully replaced with new prewarmed medium. Since the expression of α-SMA is a classical marker to positively identify HSCs upon culturing, we examined the expression of α-SMA of the harvested cells by immunofluorescence with the α-SMA antibody (CST, Catalog#19245, 1:500). In addition, to distinguish the HSCs from the hepatic myofibroblasts which also express α-SMA, we also detected the expression of desmin and glial fibrillary acidic protein (GFAP), which have been reported to be almost absent in myofibroblasts [27–29], by immunofluorescence with desmin antibody (Abcam, Catalog#ab32362, 1:500) and GFAP antibody (Abcam, Catalog#ab7260, 1:500). We observed the expression of those markers under the confocal microscopy (Leica TCS SP5) and found that almost all (>90%) of the harvested cells co-expressed the markers of α-SMA, desmin and GFAP. The HSCs used in the following experiments were all cultured for 3 days from cell attachment.

For IL-6 treatment experiments, the primary HSCs (1×10^6^ cells/plate in 6-cm dish) from the normal mouse livers were respected treated with 0, 20 and 40 ng/L of recombinant murine IL-6 (Peprotech, Catalog#216-16) for 24 hours. As for salvianolic acid B (Sal B) treatment experiments, the primary HSCs from the fibrotic mouse livers were treated with 100 ug/L of Sal B (Sigma, Catalog#49724) for 0, 3 and 6 days, followed by cell collection and RNA isolation. In the Sal B gradient experiment, the primary HSCs from the fibrotic mouse livers were respectively treated with 0, 80 and 160 ug/L for 3 days, followed by cell collection and RNA isolation. All the experiments were performed in triplicate.

### Immunofluorescence microscopy

For the EDU incorporation experiment, iClick™ EdU Andy Fluor™ 647 Imaging Kit (GeneCopoeia, Catalog#A006) was used. The experiment procedure was performed according to the manual of the kit. Firstly, the HSCs (transfected with hsa_circ_0004018 overexpressing or control plasmids) were seeded with the density of 2×10^4^ cells/cm^2^ in the 12-well culture plates with poly-D-lysine-coated glass coverslips on the bottom. After cell attachment, the medium of those for EDU incorporation experiment was replaced with EDU containing (10μM of the final concentration) new HSC culture medium. After incubation for 24 hours, the coverslips were collected and fixed with 4% paraformaldehyde, followed by DAPI staining and washing steps.

As for the detection of α-SMA and COL1A1 expression, the coverslips seeded with the primary HSCs were harvested and fixed in 4% paraformaldehyde, followed by the IgG (CST, Catalog#2729) blocking step. Then, those IgG-blocked coverslips were stained with α-SMA antibody (CST, Catalog#19245) or COL1A1 antibody (CST, Catalog# 39952) with the dilution of 1: 500, followed by secondary antibody incubation, DAPI staining and washing steps.

All the coverslips mentioned above were inverted onto the glass slides with mounting media and carefully observed and photographed under confocal microscopy (Leica TCS SP5).

### Biotin labeled probe pull down assay

The pull assay was referred to the previous study [30]. The biotin labeled hsa_circ_0004018 probe and hsa-miR-660-3p probe were synthesized by GeneChem. The sequence of hsa_circ_0004018 probe is as follows: 5’-CAGACTGGTCTTGACACTCCAATTCTCTGCCTA-3’-biotin.

The sequence of hsa-miR-660-3p probe is as follows: 5’-ACCUCCUGUGUGCAUGGAUUA-3’-biotin.

For purification, about 1×10^7^ cells were collected and lysed in lysis buffer. After that, 3μg biotin labeled probe was added to the buffer and incubated at room temperature for 4 hours. To pull down the cirRNA-microRNA complex, strepavidin magnetic beads (Thermo Fisher, Catalog#88816) were added to the buffer and slowly rotated for another 4 hours, followed by washing step for 4 times. Finally, the beads were collected by centrifuge, and the binding RNA was extracted with TRIzol reagent (Thermo Fisher, Catalog#15596026) for further real-time PCR examination.

### Cell cycle analysis

We collected about 3×10^5^ HSCs of each group (hsa_circ_0004018 overexpression vs. vector control in triplicate) for the staining of propidium iodide according to the manufacture protocol of the cell cycle analysis kits (Beyotime, Catalog#C1052). The prepared cell suspension was analyzed by FACS (BD LSRFortessa). All the FACS data were re-analyzed by FlowJo software.

### Real-time PCR

The real-time PCR experiments were performed according the manufacture protocol (GoTaq qPCR System, Promega, Catalog#A6001) on the ABI 7500 Real-Time PCR System. Each sample were examined in triplicate from three independent experiments. The PCR primers used in this study are as follows:

**Table:** 

**hsa_circ_0004018**	(Forward: 5’-GAGGTCTCAATAT GTTGCCCAGACTG-3’; Reverse: 5’-GTAGTGGACGCTTGGAAGA ATTTGGG-3’);
**α-SMA**	(Forward: 5’-GTCCCAGACATCA GGGAGTAA-3’;
	Reverse: 5’-TCGGATACTTCAGC GTCAGGA-3’);
**COL1A1**	(Forward: 5’-GAGGGCCAAGAC GAAGACATC-3’;
	Reverse: 5’-CAGATCACGTCAT CGCACAAC-3’);
**TEP1**	(Forward: 5’-CCACCCTCTCTAG TCTAAAGAGC-3’;
Reverse: 5’-CAGCTTGCGTCATG TGAGATA-3’);
**β-Actin**	(Forward: 5’-CCACCCTCTCTAG TCTAAAGAGC-3’;
	Reverse: 5’-CTCCTTAATGTCAC GCACGAT-3’).

### Western blotting

We collected the same number of cells from each group. Cells were extracted with 1% SDS cell lysis/loading buffer. All the protein samples were subjected to electrophoresis by SDS-PAGE method and then transferred to PVDF membrane for further immunoblot. The primary antibodies used in this study include: α-SMA antibody (CST, Catalog#19245, 1:1000), COL1A1 antibody (CST, Catalog# 39952, 1:1000), TEP1 antibody (Abcam, Catalog#ab64189, 1:1000), β-actin antibody (CST, Catalog#3700T, 1:3000). The blots were exposed using chemiluminescence and photographed by Tannon 3500 Imager. Photos were analyzed by ImageJ software.

### Cell proliferation assay (CCK8 assay)

For this assay, we cultured 1×10^3^ cells per wells on 96-well plates and examined the cell numbers in each well using the Cell Counting Kit (CCK8, Dojindo, Catalog#CK04) at 24, 48 and 72 hours. Each group of cells was examined in triplicate. The OD value was determined by Microplate Absorbance Reader (Bio-rad, iMark^TM^).

### Luciferase reporter assay

This assay was referred to the protocol of the previous study [31]. A 200bp fragment of hsa_circ_0004018 3’ terminal or TEP1 3’UTR was inserted into the 3’ terminal of the luciferase gene in pGL3 vector (pGL3-3’) and sequenced before use. About 1×10^5^ cells were seeded in 48-well plate before transfection. After attachment, the cells of each well were respectively transfected with 320 ng of pGL3-3’ and 30 ng of pRL-TK containing Renilla luciferase plus hsa-miR-660-3p mimic (original sequence: 5’-ACCUCCUGUGUGCA UGGAUUA-3’), mimic negative control or inhibitor (GenePharma) using Lipfatamin 2000 (Invitrogen, Catalog#11668019). 48 hours after transfection, the relative luciferase absorbance value of each group was examined by Dual-Luciferase Reporter Assay System (Promega, Catalog#E1910) and normalized to Renilla luciferase absorbance value. Each group was performed in triplicate from three independent experiments.

### Lentivirus *in-vivo* administration

The lentivirus expressing hsa_circ_0004018 or negative control was purchased from GeneChem. Lentivirus solution was injected via the tail vein (about 5×10^8^ units of each mouse) twice respectively at the first and the second week of CCl_4_ injection (once a week for 4 weeks). All groups of mice were sacrificed at the end of 4 weeks for further pathological examination.

### Immunochemistry

The liver tissue samples collected from the model and the control mice were fixed in 4% paraformaldehyde overnight at room temperature. Following with the steps of dehydration, embedding in paraffin, the samples were sliced into 5-8μm thickness and transferred onto glass slides. The sections were then immunostained with α-SMA and COL1A1 primary antibody at 4 °C overnight and then incubated with biotinylated secondary antibody. After incubation with Sav-HRP conjugates, the sections were applied with DAB substrate for color development and observed under microscopy.

### CCl_4_-induced liver fibrosis mouse model

The liver fibrosis mouse model was established referring to the previous report [32]. C57BL/6 male mice of 6-8 weeks old and 20±2 g in weight were supplied by the Experimental Animal Center of Hubei University of Medicine. Each mouse was treated with 2 ml of CCl_4_ (Sinopharm Chemical Reagent Co., Catalog# 10006480)/olive oil (1:1, v/v) per kg body weight by intraperitoneal injection twice for 6 weeks. Mice injected with equal volume of olive oil were performed as control.

All the mice used in this study were under human care and provided with enough food and water. When it came to the end of each experimental point, those mice were euthanatized with CO_2_. All the experiments were approved and supervised by the Animal Welfare and Ethics Committee of Renmin Hospital, Hubei University of Medicine.

### Masson’s trichrome staining and the morphometry

The liver samples were isolated from the liver fibrosis mice sacrificed on day 0, 30 and 45 upon injection with CCl_4_ and then fixed in 4% paraformaldehyde and embedded in paraffin, followed by frozen section. For the Masson’s trichrome staining of the liver sections, Trichrome Stain Kit (Abcam, Catalog#ab150686) was used. Briefly, the sections were firstly deparaffinized and rehydrated in distilled water. Then, the sections were incubated successively with preheated Bouin's Fluid, Weigert's Iron Hematoxylin, Biebrich Scarlet/Acid Fuchsin solution, phosphomolybdic/phosphotungstic acid solution, Aniline Blue solution and acetic acid solution, with rinse steps at the interval of every two incubation steps. The duration of each incubation step was controlled according to the recommendatory procedures of the manual. The stained sections were then carefully observed and photographed under microscope (Olympus). The proportion of each staining area was analyzed by ImageJ software. The blue staining area represents the collagen-enriched tissues, which was considered as the fibrosis area of the livers.

### Statistical analysis

All the data were analyzed using SPSS software. Student’s t test was used to compare the difference of data between groups. P<0.05 represents significant difference. The asterisks *, ** and *** stand for p<0.05, p<0.01 and p<0.001, respectively.

### Data availability statement

The datasets used in the current study are available from the corresponding author on reasonable request.
