# Correction for: hsa_circ_0004018 suppresses the progression of liver fibrosis through regulating the hsa-miR-660-3p/TEP1 axis

**DOI:** 10.18632/aging.203218

**Published:** 2021-06-15

**Authors:** Shan Li, Fangmin Song, Xu Lei, Jingtao Li, Fang Li, Huabing Tan

**Affiliations:** 1 Department of Infectious Diseases and Lab of Liver Disease, Renmin Hospital, Hubei University of Medicine, Shiyan, Hubei, China; 2 Department of Infectious Diseases, People’s Hospital of Yunxi, Shiyan, Hubei, China; 3 Department of Liver Diseases, The Affiliated Hospital of Shaanxi University of Chinese Medicine, Xianyang, Shaanxi, China

**Keywords:** correction

Original article: Aging. 2020; 12:11517–11529.  . https://doi.org/10.18632/aging.103257

**This article has been corrected:** The authors replaced panel 7G, representing expression of α-SMA in liver samples of the cirRNA group and the NC group, by using the representative images from the original set of experiments. This correction does not affect the results or conclusions of this work.

**Figure 7:**
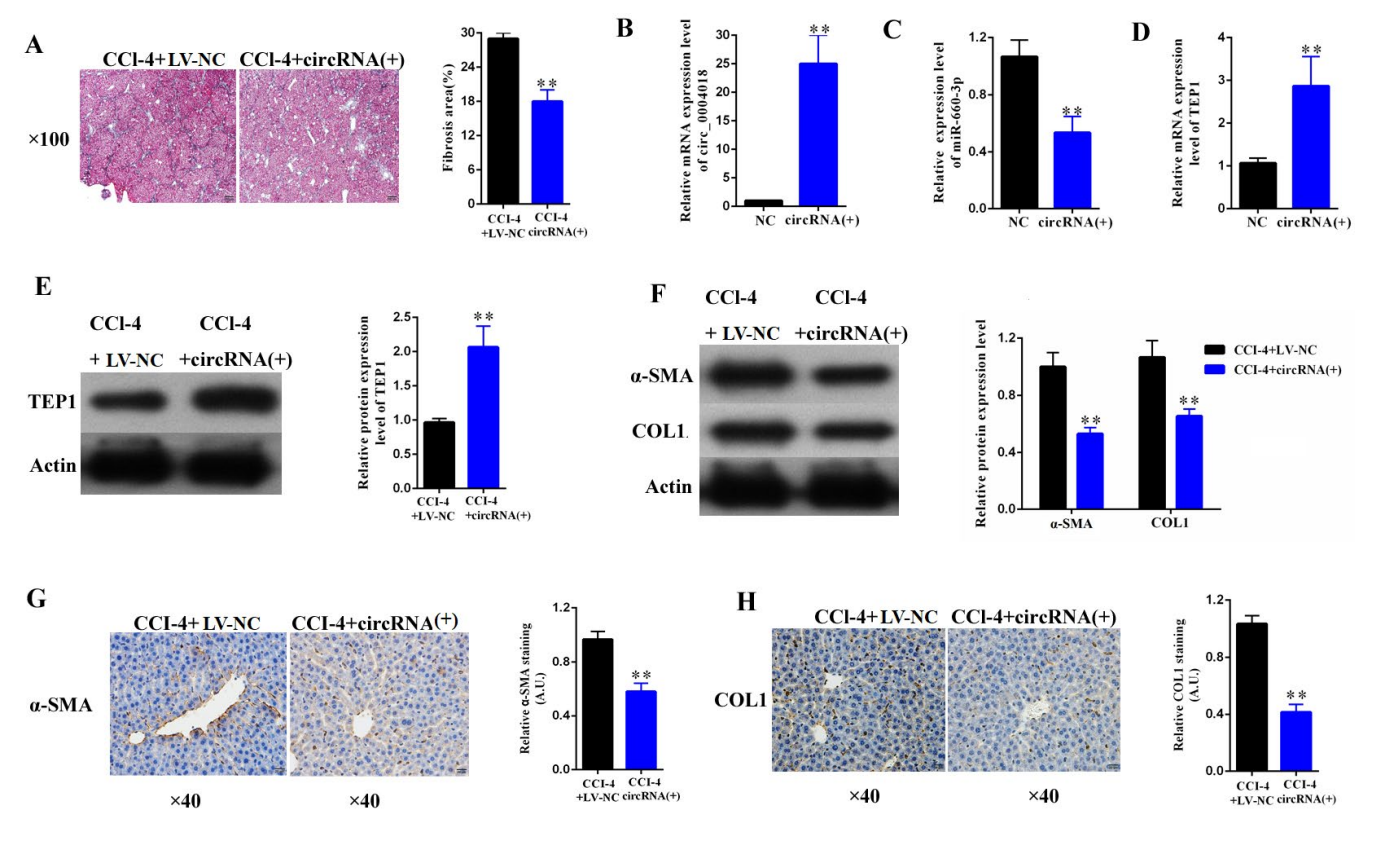
**Overexpression of hsa_circ_0004018 alleviated CCl4-induced mouse liver fibrosis through suppressing the expression of hsa-miR-660-3p and upregulating TEP1 in HSCs.** (**A**) The representative section staining of the liver samples isolated from the CCl4-induced liver fibrosis mice respectively injected with lentivirus expressing hsa_circ_0004018 (cirRNA group) or the negative control (NC group). (**B, C**) They were detected by real-time PCR the RNA levels of hsa_circ_0004018 (**B**) and hsa-miR-660-3p (**C**) in the liver samples of the cirRNA group and the NC group mice. (**D, E**) The RNA levels (**D**) and the protein levels of TEP1 (**E**) were respectively detected in the liver samples of the cirRNA group and the NC group mice. (**F–H**) It was detected the expression of α-SMA and COL1A1 in liver samples of the cirRNA group and the NC group by western blotting (**F**) and immunohistochemistry (**G, H**), respectively.

